# Diagnostic pitfalls for *GJB2*‐related hearing loss: A novel deletion detected by Array‐CGH analysis in a Japanese patient with congenital profound hearing loss

**DOI:** 10.1002/ccr3.1800

**Published:** 2018-09-21

**Authors:** Satoko Abe, Shin‐ya Nishio, Yoh Yokota, Hideaki Moteki, Kozo Kumakawa, Shin‐ichi Usami

**Affiliations:** ^1^ Department of Otorhinolaryngology Toranomon Hospital Tokyo Japan; ^2^ Department of Otolaryngology Shinshu University School of Medicine Nagano Japan; ^3^ Department of Hearing Implant Sciences Shinshu University School of Medicine Nagano Japan

**Keywords:** CNV, *GJB2* gene, novel deletion, profound hearing loss

## Abstract

Here, we report a novel deletion (copy number variation: CNV) in the *GJB2* gene observed in a Japanese hearing loss patient. The deleted segment started in the middle of the *GJB2* gene, but the *GJB6* gene remained intact. This partial deletion in the *GJB2* gene highlights the need for further improvements in *GJB2* screening.

## 1 INTRODUCTION

*GJB2* gene mutations are known to be the most common cause of hereditary hearing loss worldwide. Therefore, genetic testing for *GJB2* mutations is one of the most important screening processes for the molecular diagnosis of deafness. However, some caution is due in the diagnosis of hearing loss based on GJB2 screening as several types of large deletions have been reported. Here, we report a novel deletion (copy number variation: CNV) in the *GJB2* gene observed in a Japanese patient presenting with profound hearing loss. This deletion was observed in *trans* to a *GJB2*‐mutated allele carrying the *GJB2*:NM_004004.5:c.427C>T:p.R143W mutation by PCR fragment analysis. It should be noted that this deletion was identified as homozygosity of c.427C>T:p.R143W by Sanger sequencing. Array‐CGH analysis showed the deleted segment started in the middle of the *GJB2* coding region and extended for at least eight thousand base pairs, although the *GJB6* gene remained intact. The distal breakpoint downstream of the *GJB6* gene differed from the breakpoints of the known DFNB1 locus deletions. This partial deletion in the *GJB2* gene highlights the need for further improvements in *GJB2* screening.

Inherited sensorineural hearing loss (HL) is an extremely heterogeneous group of sensory disorders in humans. The overall incidence is estimated to be one in approximately 1000 newborns.^1^, ^2^ The *GJB2* gene (MIM# 121011), which encodes the gap junction protein connexin 26 (Cx26), is the most common genetic etiology associated with congenital HL worldwide, with the mutation spectrums known to vary among different ethnic groups.^3^, ^4^, ^5^ The *GJB2* gene is a small gene composed of two exons, one of which possesses a 678‐bp coding sequence. As screening of the *GJB2* gene is considered a standard, first‐step approach in the diagnosis of genetic hereditary HL, it is not surprising that more than 300 mutations in the *GJB2* gene sequence have been described (The Human Gene Mutation Database). In general terms, *GJB2*‐related congenital HL develops through a biallelic mutation. Among patients with a single *GJB2* heterozygous status, some caution is due in the diagnosis of hearing loss based on GJB2 screening as a large deletion located in the 13q12 region encompassing the *GJB2* and *GJB6* genes (the so‐called DFNB1 locus) is sometimes seen in *trans* with *GJB2*‐coding region variants. Therefore, the potential for such deletions to be present should be kept in mind. To date, six large deletions contributing to HL have been identified in the DFNB1 region.^6^, ^7^, ^8^, ^9^, ^10^, ^11^

Here, we report for the first time a novel large deletion in the *GJB2* gene in one Japanese family with nonsyndromic HL. The present work highlighted the diagnostic pitfalls of GJB2‐related hearing loss and could expand the pathogenic spectrum and strengthen our understanding of the complicated mechanisms by which *GJB2* gives rise to HL.

## 2 CLINICAL REPORT

### 2.1 Ethics statement

This study was approved by the Ethical Committee for Clinical Research of Shinshu University and each participating institution as described previously. We obtained written informed consent from all the participants (or their parents) involved in this study.

**Figure 1:**
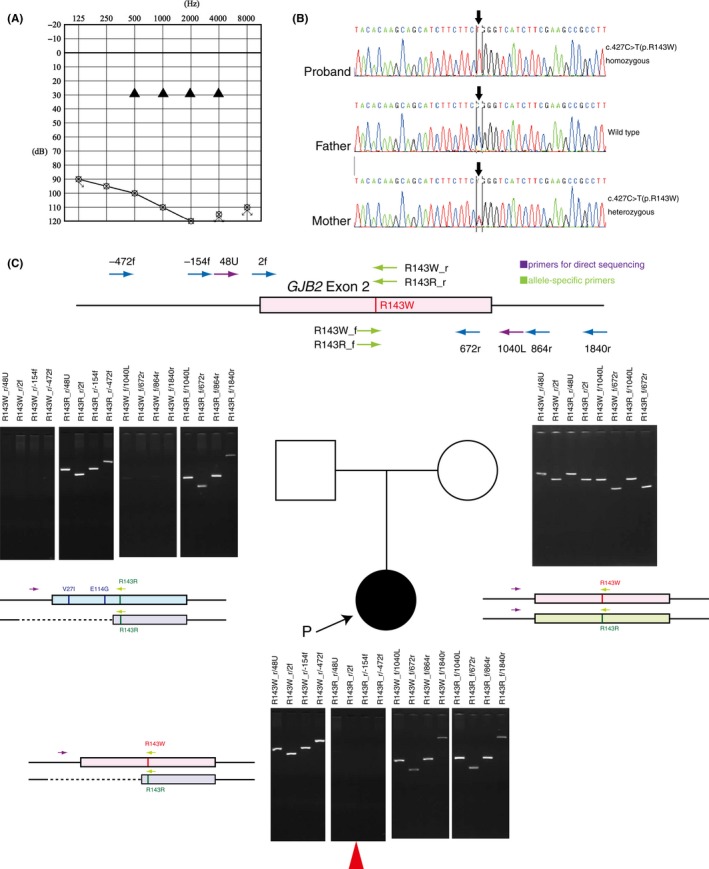
Pedigree and genomic DNA analysis. A, The audiograms of both ears in the proband of the family revealed profound hearing loss. Black triangles: the hearing threshold level via cochlear implant. B, Direct sequencing electropherograms of *GJB2* showing the p.R143W homozygote in the patient. C, Agarose gel electrophoresis of the PCR products in this family. In the patient and her mother, p.R143W‐specific amplified bands are observed. PCR products for p.R143R_r allele‐specific and upstream designed primers were absent (red arrowhead) in the patient only. For the wild‐type sample, all the corresponding amplified PCR fragments were observed except for PCR amplification by p.R143W‐specific primers

One two‐generation Japanese family with sporadic nonsyndromic sensorineural HL was introduced to the Department of Otolaryngology at the Toranomon Medical Hospital. The patient of the family was a 3‐year‐old girl who was referred for newborn auditory screening in both ears: Her wave V at 90 dB nHL did not show a bilateral response during auditory brainstem response testing at 5 weeks after birth. Her auditory steady‐state response (ASSR) examination showed thresholds of 95‐115 dB for the right ear and 115 dB for the left, indicating profound hearing loss. Conditioned orientation response (COR) testing showed reactions in the range of 90‐100 dB, while her tympanometric results were normal. After confirmation of hearing loss, the patient started wearing hearing aids in both ears, but showed only limited audiological improvement and difficulty with language acquisition. Pure‐tone audiometry at four frequencies (0.5, 1, 2, and 4 kHz) showed a bilateral configuration characteristic of profound hearing loss (>95 dB HL) (Figure 1A). At 3 years and 6 months of age, the patient underwent cochlear implantation in her left ear. She has since showed steady progress in language development as her ability to recognize words has increased dramatically. Her average hearing threshold (0.5, 1, 2, and 4 kHz) is 30 dB when wearing the cochlear implant (Figure 1A). Her development was normal, and no other phenotypic abnormalities, apart from left fistula auris congenita, were noted. Her parents have no hearing impairment. Screening for congenital cytomegalovirus infection using her preserved umbilical cord specimen was negative for cytomegalovirus DNA. Temporal bone computed tomography (CT) scans also revealed no abnormalities.

### 2.2 DNA analysis

Independent of PCR‐based methods, the proband's Invader assay results showed a *GJB2*:c.427C>T:p.R143W heterozygous pattern at both 2 and 4 hours (data not shown). Next, we considered that a second *GJB2* mutation could be present in the other allele as the *GJB2* gene can cause prelingual HL due to biallelic mutations. Sanger sequencing for the family members revealed that, quite unexpectedly, the affected girl had the *GJB2*:c.427C>T:p.R143W homozygote (Figure 1B), and the mother of the patient, but not the father, carried a p.R143W heterozygous mutation. Sanger sequencing also revealed that the *GJB2*:c.427C>T:p.R143W variant did not segregate with the phenotype and that it was inconsistent with the results of the Invader assay. Genotypes of two nonpathogenic variations (V27I and E114G, which are located upstream of *GJB2*:c.427C>T:p.R143W) also showed similar segregation in this family (data not shown). As the results of both the Invader assay and Sanger sequencing are highly reliable, as part of our molecular interpretation of these discrepancies, we hypothesized that a paternally transmitted deletion in the *GJB2* gene might exist and that only one of the two alleles might be read in the direct sequence. To verify our hypothesis, we performed DNA assay with uniquely designed PCR primers sets including the R143R and R143W positions (allele‐specific primers). In her mother, both of the allele‐specific primers for the wild type and R143W mutation were amplified and PCR products of the expected size were confirmed as one band. For her father, no fragments were detected for R143W‐specific PCR amplification, indicating an apparently wild‐type sequence. For the proband, PCR products were obtained with the R143W‐specific primers in both the upstream and downstream regions. On the other hand, no allele‐specific PCR product containing R143R‐r was amplified, even outside the −472‐bp region upstream from exon 2 (Figure 1C); however, a R143R‐f product was obtained.

**Figure 2:**
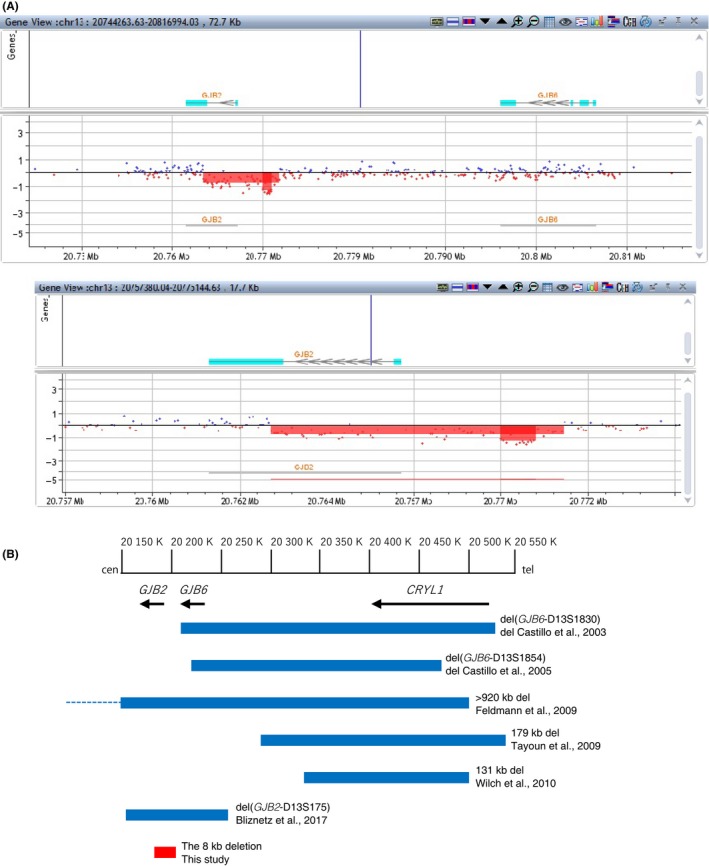
Location of deletions at the DFNB1 locus on chromosome 13q12.11. A, Results of Array‐CGH for the *GJB2* gene region in the patient. Hybridization results are shown for the affected patient in whom an abnormality was identified by Array‐CGH. For each panel, the *x*‐axis marks the distance, in megabases, along the chromosome from the q telomere. The *y*‐axis marks the hybridization ratio plotted on a log_2_ scale. The patient shows a hemizygous deletion involving in the *GJB2* gene region based on analysis using CytoGenomics software. The area shown in red is depleted by one copy loss. The upper panel includes the region up to *GJB6*, and the lower panel is an expanded image showing the approximate eight thousand base pair deletion. B, Schematic map of seven deletions. All genomic configurations are derived from NC_000013.11(Reference: GRCh38.p12 Primary Assembly)

These results clearly indicated the long deletion was located in the region upstream of the *GJB2*:p.R143 site in a single allele. Array‐CGH was then undertaken as described previously.^12^ The Array‐CGH profiles for the aberrant 13q12 region are shown in Figure 2A. A deletion of approximately eight thousand base pairs (8 kb) from the *GJB2* gene region was identified by Array‐CGH, but this deletion did not extend to the *GJB6* gene. This novel deletion starts from the middle of the *GJB2* coding region sequence and spreads upstream of *GJB2*, differing from the breakpoint of the other known large DFNB1 locus deletions (Figure 2B). The deletion region deduced from the Array‐CGH probe position was Chr13: 20763410‐20771801 in GRCh37.5. Finally, we concluded that the HL in this patient was caused by a compound heterozygous mutation of *GJB2*:c.427C>T:p.R143W with a long deletion and was passed down by autosomal recessive inheritance.

## 3 DISCUSSION

In this study, we identified a novel deletion in the *GJB2* gene (hereafter, the 8 kb deletion), in *trans* with a p.R143W heterozygous status, in a Japanese patient with profound HL. Our DNA fragment analysis by PCR amplification indicated the existence of a broad deletion that appears to possess a breakpoint starting between p.E114 and p.R143 in the *GJB2* coding region and extending upstream. Array‐CGH was next implemented, revealing an overview of the new deletion (Figure 2B). We calculated the deletion to be approximately 8 kb in length, but that it did not affect the *GJB6* gene. In this work, we detected for the first time the presence of a single large deletion in the *GJB2* gene sequence. The long deletion identified in the *GJB2* gene in this study was not identified in the DECIPHER databases (https://decipher.sanger.ac.uk) or our 154 in‐house controls (data not shown). The identified *GJB2* deletion region contains the translational start codon, 5′ UTR, and a transcriptional promoter region of the *GJB2* gene. The absence of the transcriptional promoter sequence is thought to abolish *GJB2* gene transcription. Previous functional studies of c.‐22‐2A>C (a splice‐site mutation) and g.‐77C>T (a mutation of the basal promoter region) did not yield any detectable Cx26 protein or mRNA,^13^, ^14^ suggesting that no *GJB2* transcripts would be produced from this deletion allele. The proband's father, harboring a heterozygous 8 kb deletion, is not conscious of any hearing loss, implying that the novel long deletion is a pathogenic variant in autosomal recessive inheritance HL. The p.R143W variant shows an extraordinarily high prevalence among congenital HL patients in Ghana, West Africa.^4^ Not limited to Africa, it is also commonly found in congenital HL patients among various populations.^5^, ^15^ Biallelic‐mutated individuals with R143W and *GJB2* truncating mutations display profound HL.^5^, ^15^ Considering the 8 kb deletion to be equivalent to a truncating mutation, there appears to be no contradiction in the fact that this patient shows a profound hearing impairment phenotype. According to several studies on the carrier frequency of *GJB2* mutations, the p.R143W allele frequency is 0.42%(13/3062 alleles) ~0.67% (18/2686 alleles) in congenital HL patients among different ethnic groups,^5^, ^15^ and a frequency of up to 0.1% (1/1018 alleles) was observed in the general Japanese population.^16^ The p.R143W mutation is mostly found in a compound heterozygous form, except for in Ghana. As p.R143W is a pathologic variation of relatively rare frequency (<1%), apart from inbred or familial HL accumulation, it is unlikely to occur in a homozygous p.R143W genotype. In genetic counseling of recessive families or simplexes in which a *GJB2* homozygous mutation is detected, it is generally explained as parental heterozygous inheritance or uniparental disomy of chromosome 13, but the latter is extremely uncommon.^17^, ^18^ Thus, when possible, segregation studies are needed to confirm whether both parents are carriers or not. Sanger sequencing, by overlooking a large deletion in the DFNB1 locus, may lead to a judgment of apparent *GJB2* homozygosity.^8^ Even though the probability is very small, to avoid missing such large deletions in the DFNB1 locus, other DNA tests capable of detecting large deletions should be used in combination for screening the *GJB2* gene. Recently targeted next‐generation sequencing (NGS) has been applied to the high throughput and fast identification of pathological causes.^19^ In addition, the NGS strategy can be expected to provide an effective method for the identification of multi‐exon deletions or duplications of target genes.^20^ Therefore, smooth progression to the analysis of gene dosage for confirmation may bring about precise diagnosis and appropriate genetic counseling for *GJB2*‐related HL without the need for Sanger sequencing. Although the additional analysis may be required for further refinement of this novel deletion, the results of our study have furthered our knowledge of GBJ2 and its pathogenicity.

## CONFLICT OF INTEREST

The authors have no conflicts of interest to declare.

## AUTHOR CONTRIBUTIONS

SA, SN, and SU: wrote the manuscript. SA, KK, and HT: collected the detailed clinical findings of patients and obtained DNA samples. SN: performed allele‐specific PCR analysis. YY, HM, and SN: performed aCGH analysis.
